# Probing Bunyavirus N protein oligomerisation using mass spectrometry

**DOI:** 10.1002/rcm.6841

**Published:** 2014-02-17

**Authors:** Dale A Shepherd, Antonio Ariza, Thomas A Edwards, John N Barr, Nicola J Stonehouse, Alison E Ashcroft

**Affiliations:** Astbury Centre for Structural Molecular Biology, Faculty of Biological Sciences, University of LeedsLeeds, LS2 9JT, UK

## Abstract

**RATIONALE:**

Bunyaviruses have become a major threat to both humans and livestock in Europe and the Americas. The nucleocapsid (N) protein of these viruses is key to the replication cycle and knowledge of the N oligomerisation state is central to understanding the viral lifecycle and for development of therapeutic strategies.

**METHODS:**

Bunyamwera virus and Schmallenberg virus N proteins (BUNV-N and SBV-N) were expressed recombinantly in *E. coli* as hexahistidine-SUMO-tagged fusions, and the tag removed subsequently. Noncovalent nano-electrospray ionisation mass spectrometry was conducted in the presence and absence of short RNA oligonucleotides. Instrumental conditions were optimised for the transmission of intact protein complexes into the gas phase. The resulting protein-protein and protein-RNA complexes were identified and their stoichiometries verified by their mass. Collision-induced dissociation tandem mass spectrometry was used in cases of ambiguity.

**RESULTS:**

Both BUNV-N and SBV-N proteins reassembled into N-RNA complexes in the presence of RNA; however, SBV-N formed a wider range of complexes with varying oligomeric states. The N:RNA oligomers observed were consistent with a model of assembly via stepwise addition of N proteins. Furthermore, upon mixing the two proteins in the presence of RNA no heteromeric complexes were observed, thus revealing insights into the specificity of oligomerisation.

**CONCLUSIONS:**

Noncovalent mass spectrometry has provided the first detailed analysis of the co-populated oligomeric species formed by these important viral proteins and revealed insights into their assembly pathways. Using this technique has also enabled comparisons to be made between the two N proteins.

The *Bunyaviridae* family of segmented, negative sense, single-stranded RNA viruses comprises five genera: *Hantavirus*, *Nairovirus*, *Phlebovirus*, *Tospovirus* and *Orthobunyavirus*. Amongst the 330-plus viruses in the *Bunyaviridae* family are several human pathogens, including Rift Valley fever virus (RVFV, phlebovirus), Crimean-Congo haemorrhagic fever virus (CCHFV, nairovirus) and Sin Nombre virus (SN, hantavirus). Studies of these viruses are particularly timely, given the migration of many arthropod vectors into northern Europe as a result of changing climate.^1^

In 2011, a novel bunyavirus was isolated in Europe and found to cause severe birth defects and abortion in livestock such as sheep, cattle, and goats.^2^ The virus was termed Schmallenberg virus (SBV) and classified in the genus *Orthobunyavirus*, the largest of the family. This genus also contains Bunyamwera virus (BUNV), the prototypic member of the family.

The orthobunyavirus genome consists of three separate strands of negative-sense single-stranded RNA. These are termed the large (L), medium (M), and small (S) segments and are packaged into viral ribonucleoprotein complexes (RNPs) incorporating the nucleocapsid protein (N). The N protein protects the viral genome and is essential for the viral replication cycle; the viral RNAs are only able to participate in transcription and replication when complexed with N as RNPs, and only intact RNPs are packaged into virions.^3^ The L segment mRNA codes for the viral RNA-dependent RNA polymerase (RdRp),^4^ the M segment mRNA codes for the surface glycoproteins Gn and Gc together with a non-structural protein (NSm)^5^ and the S segment mRNA codes for the N protein in addition to the non-structural NSs protein, with which it has an overlapping reading frame.^6^

Recently, there have been several advances in the field of orthobunyavirus structural biology, with the crystal structures of the N proteins from four different viruses, namely SBV,^7^–^9^ BUNV,^7^,^10^ Leanyer virus (LEAV),^11^ and the human pathogen, LaCrosse virus (LACV),^12^ being solved independently by several groups. These structures display a novel, highly conserved fold, with a common oligomerisation interface mediated by flexible N- and C-terminal arms (Fig. 1).

**Figure 1:**
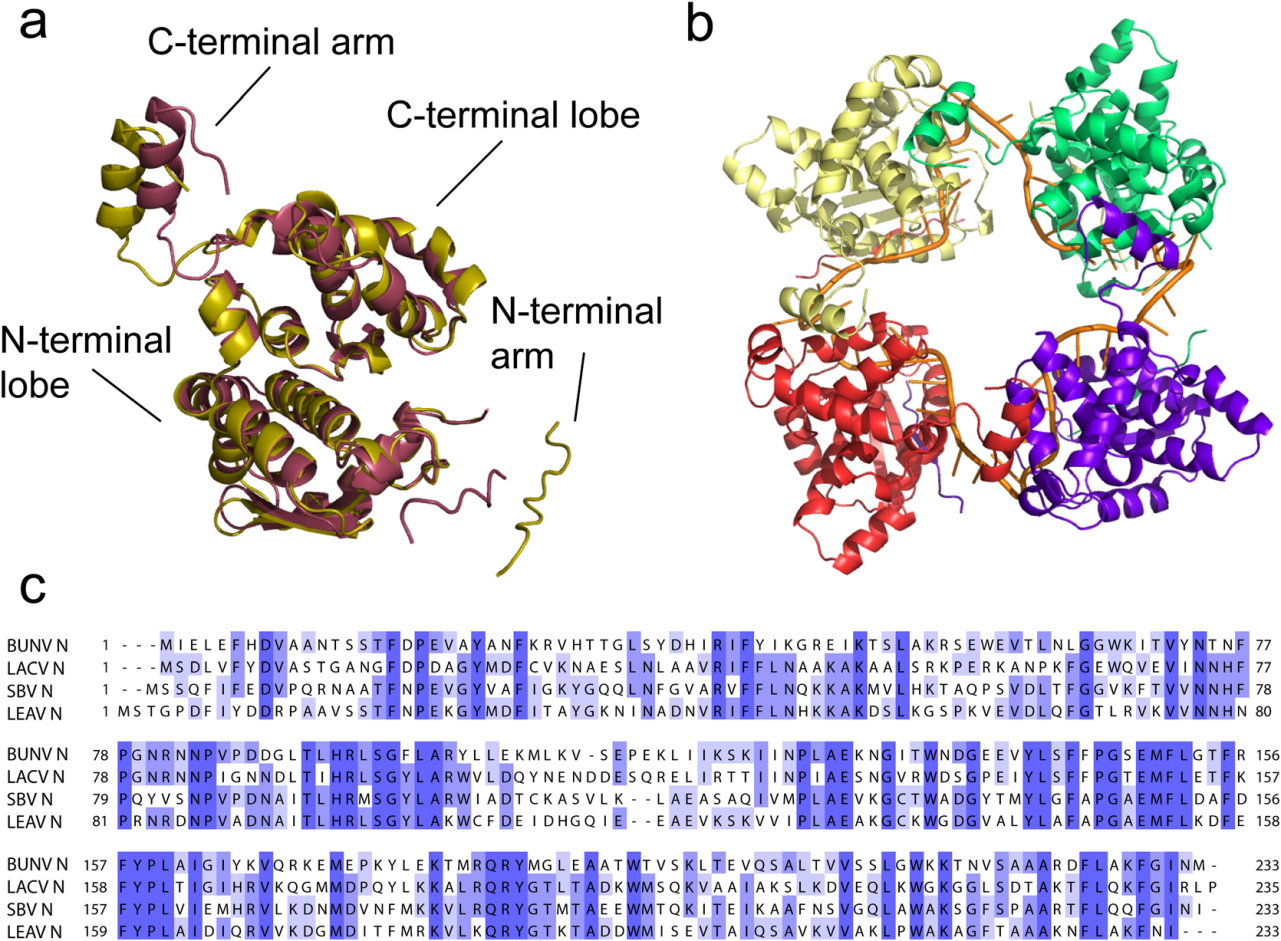
The crystal structures of BUNV-N and SBV-N. (a) Overlay of BUNV-N (yellow, PDB ID 3ZLA) and SBV-N (maroon, PDB ID 3ZL9) monomer crystal structures taken from the crystallographic tetramers, illustrating similarities in the fold; (b) BUNV-N tetramer bound to RNA (orange stripe); (c) sequence alignment of the N proteins of BUNV, SBV, LACV, and LEAV. Degree of conservation (percentage identity) is indicated in shades of blue.

Previously, we have conducted structural studies on both SBV-N and BUNV-N.^7^ BUNV-N was crystallised as a tetramer bound to RNA whereas SBV-N was crystallised as an RNA-free tetramer. The folds were shown to be very similar despite only 37% sequence identity between the proteins (Fig. 1(a)). The RNA-bound structure of BUNV-N^7^ and that of the other orthobunyaviruses^9^–^12^ provided unambiguous determination of the RNA-binding groove. The tetramer was bound to 44 nucleotides (nt) (as for LEAV^11^ and LACV^12^), i.e. 11 nt per N monomer. The BUNV-N RNA was shown to bind in a highly electropositive groove with bases packed deep in the groove^7^,^9^–^12^ (Fig. 1(b)).

With the establishment of the crystal structures of these orthobunyavirus N proteins came a significant leap forward in the knowledge of the architecture of these viruses; however, many of the details of the RNP superstructure and RNA-binding properties remained elusive. To date, orthobunyavirus RNPs have been shown by electron microscopy (EM) as circular, loose, string-like particles; however, the results of observations of the width of the 'strings' have varied. Our previous EM data and those of Reguera *et al*. show agreement between the width of the orthobunyavirus N tetramer and the width of intact RNPs isolated from infectious virions,^7^,^12^ whereas data from Li *et al*.^10^ and Niu *et al*.^11^ report an RNP width more in line with the size of a monomer. The discrepancies between these studies may indicate that both expanded and condensed forms of the RNP can exist,^7^,^13^ and that these could have distinct roles during the viral replication cycle. A loose helical geometry would clearly support both types of RNP and a helical crystal form has been identified for LACV with a pitch of 5.4 nm.^12^ Despite these data, much is still to be learned about the orthobunyavirus N assembly process and RNA-binding properties. The relationship between the N monomer and tetramer is unclear, as is the role, if any, that the ring-like N tetramer plays in RNP assembly.

Electrospray ionisation mass spectrometry (ESI-MS) has been employed in a wide range of studies investigating the structure and function of protein assemblies and has been found to be particularly powerful in the study of virus systems. This technique has been used to detect and interrogate whole virus capsids,^14^–^17^ capsid assembly intermediate complexes,^18^–^20^ and viral protein organelles.^21^,^22^ Particular beneficial features of ESI-MS are its ability to transfer fragile complexes from the solution to the gas phase whilst maintaining non-covalent interactions intact, and to separate and identify individual constituents within heterogeneous populations by virtue of their mass.

In this communication, we demonstrate the use of ESI-MS to investigate the RNA-binding and oligomerisation properties of the N proteins of both the prototype BUNV and the recently emerging SBV. We rationalise the observed species and propose a simple model of assembly. For example, if a higher-order RNA-N complex is composed of stacked tetrameric rings of N proteins, we may expect to see addition of 'blocks' of tetramers of N proteins during oligomerisation. However, here we observed a step-wise addition of monomers and/or dimers of N. Also of note is the lack of a heteromeric N-N interaction between BUNV-N and SBV-N, revealed by tandem mass spectrometry (MS/MS) and collision-induced dissociation (CID), which is somewhat unexpected considering the similarity in the folds of the two proteins. We attribute these observations to differences in the sequences of the unstructured N-terminal arm, a region revealed by crystallography^7^,^8^,^11^,^12^ and mutagenesis^23^ studies to be involved in N-N interactions.

## EXPERIMENTAL

### Protein preparation

SBV-N was produced as described previously.^7^ BUNV-N was cloned expressed and purified in the same way as SBV-N with the following modification: the wash buffers during the first metal affinity chromatography step contained 25, 50, 75, and 100 mM imidazole, respectively.

### nanoESI-MS analysis

All mass spectra were acquired using a Synapt HDMS orthogonal acceleration quadrupole-travelling wave time-of-flight mass spectrometer (Micromass UK Ltd, Waters Corp., Manchester, UK). Samples were analysed using nano-ESI from platinum/gold-plated borosilicate capillaries fabricated in-house using a P-97 micropipette puller (Sutter Instrument Co., Novato, CA, USA) and a sputter coater (Polaron SC7620; Quorum Technologies Ltd, Ashford, UK). All spectra were calibrated using cluster ions generated from a separate introduction of caesium iodide solution (40 mg mL^–1^ in water). Data were analysed using the MassLynx 4.1 software package (Micromass UK Ltd, Waters Corp.). Samples of each N protein were analysed at a target protein concentration of 2.5 μM tetramer in ammonium acetate buffer (pH 6.8). Spectra were acquired using a capillary voltage of 1.2–1.4 kV, a cone voltage of 60 V, a trap collision voltage of 6 V, a transfer collision voltage of 14 V, a trap DC bias of 20 V, a trap flow rate of 3 mL min^–1^, and a source pressure of 4 mbar. MS/MS was performed by precursor mass-selection in the quadrupole analyser followed by CID in the argon-filled trap ion guide of the Synapt HDMS mass spectrometer. Trap collision voltages in the range 40–90 V were used to dissociate the protein complexes in order to verify their identities. The product ions generated were *m/z* analysed using the time-of-flight analyser.

### nanoESI-IMS-MS analysis

For ion mobility experiments, the following parameters were set on the Synapt HDMS mass spectrometer: a wave height of 11 V and a wave velocity of 300 m s^–1^. For the calculation of rotationally averaged collision cross-sectional areas, calibration of the travelling-wave ion mobility device was carried out using β-lactoglobulin A, avidin, concanavalin A, yeast alcohol dehydrogenase, pyruvate kinase, and glutamate dehydrogenase (all purchased from Sigma-Aldrich, Poole, UK).

Experimentally optimised coarse-grained structural models were built approximating each N monomer as a sphere, for comparison with the experimentally calculated collision cross-sectional areas. The radius of the spheres was set at 2.5 nm, the radius required to give a collision cross-section of 21.6 nm^2^, the experimental collision cross-sectional area measured for SBV N. The centre-to-centre distance of protein spheres in the tetramer model was optimised so that its theoretical collision cross-section was in agreement with the experimental value of tetrameric SBV N. This facilitated the building of a range of oligomer models as it gave an estimate of the buried protein surface upon N oligomerisation. Theoretical single-ring (trimers-hexamers) and double-ring (octamers, decamers, and dodecamers) structures were built using the experimentally optimised protein radius and centre-to-centre distance for comparison with experimental data. All theoretical collision cross-sections were calculated using the Leeds' Method projection approximation algorithm.^20^

### Incubation of N proteins with synthetic oligoribonucleotides

The oligoribonucleotides (RNAs) 12-mer (5′-AGUAGUGUACUC-3′, 3795 Da), 24-mer (5′-AGUAGUGUACUCCACACUACAAAC-3′, 7803 Da), 48-mer (5′-AGUAGUGUACUCCACACUACAAACUUGCUAUUGUUGAAAAUCG CUGUG-3′, 15,297 Da), and 60-mer (5′- AGUAGUGUACUCCACACUACAAACUUGCUAU UGUUGAAAAUCGCUGUGCUAUUAAAUCCA-3′ 19,082 Da) were purchased from Integrated DNA Technologies (Glasgow, UK). It was not feasible to produce longer RNAs by chemical synthesis at the purity required for these experiments. The masses of the RNAs were confirmed by negative ion ESI-MS in solutions of 50:49:1 acetonitrile/50 mM ammonium acetate/triethylamine (v/v/v).

RNAs were added to samples of either BUNV-N or SBV-N in various molar ratios as stated in the Results and Discussion section using a protein concentration that would lead to a final concentration of 10 μM, based on the monomer. MS and MS/MS were used to confirm the oligomeric states that were formed in the presence of the RNAs.

### Mixing BUNV-N and SBV-N proteins

The N proteins were mixed in a 1:1 molar ratio in 50 mM ammonium acetate (pH 6.8). The 24-mer RNA was added in a 2:1 RNA/total protein ratio. Mass spectra were acquired under the same conditions as above.

## RESULTS AND DISCUSSION

### RNA-free BUNV-N exists as a monomer

**Figure 2:**
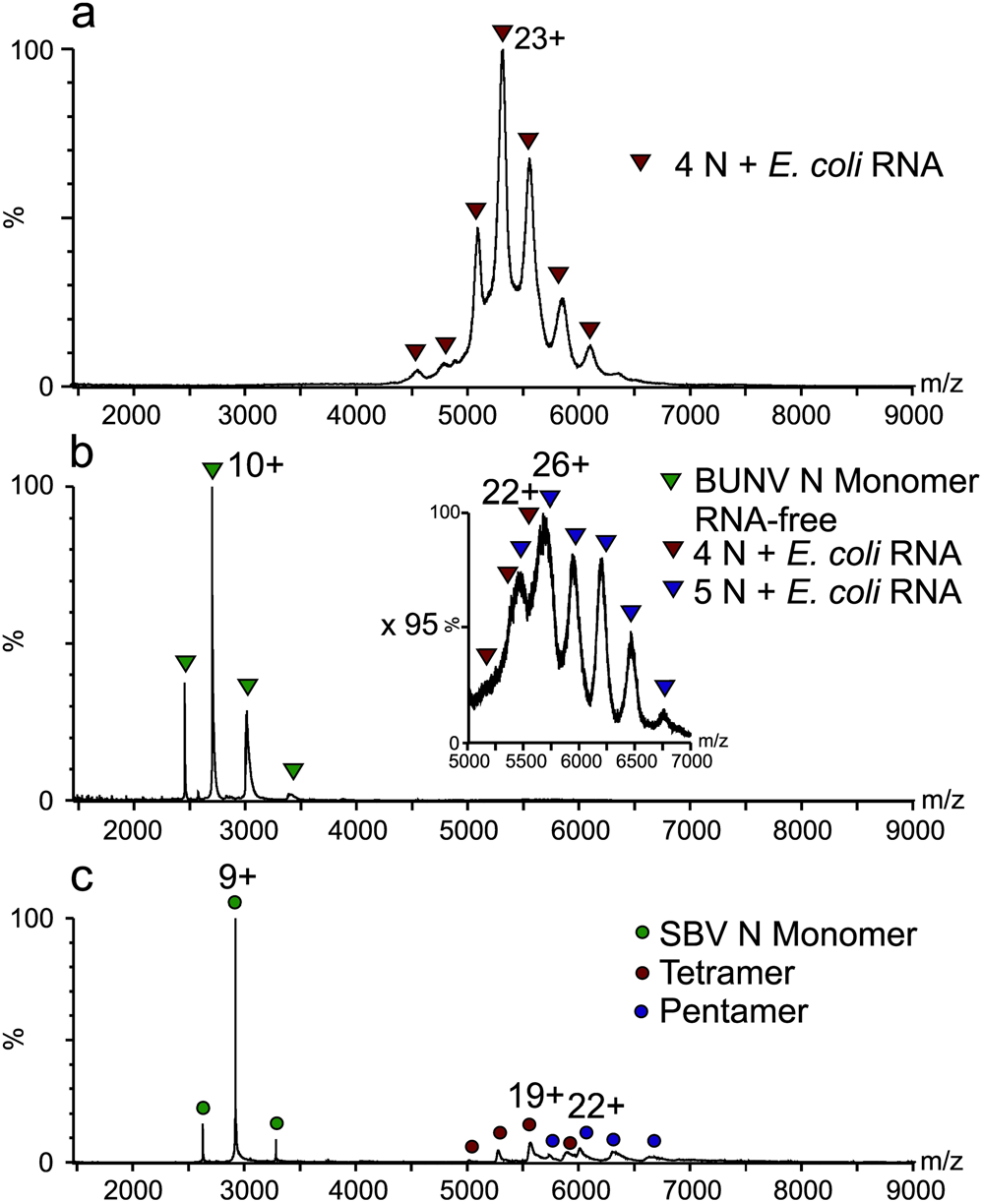
ESI-MS shows that RNA is crucial for N protein complex stability. (a) BUNV-N with endogenous *E. coli* RNA. The observed complex (20+ to 26+ charge state ions) has a mass of 120 894 Da consistent with a BUNV-N tetramer bound to 44 nt of RNA; (b) BUNV-N is predominantly monomeric (mass 26 751 Da; 8+ to 11+ charge state ions, green triangles) after removal of the endogenous RNA. Inset: expansion of *m/z* 5000–7000 region showing traces of RNA-bound tetramers and pentamers remaining after the RNA-removal step (red and navy triangles, respectively); (c) SBV-N exists as a monomer (mass 26 269 Da) (8+ to 10+ charge state ions, green circles), tetramer (*m/z* 5000–6000, red circles), and pentamer (*m/z* 5500–7000, navy circles) in the absence of endogenous RNA.

Recombinant BUNV-N has been shown previously to be purified predominantly as a tetramer bound to endogenous RNA co-purified from the *E. coli* expression host.^7^ ESI-MS of this product corroborates this (Fig. 2(a)), with a mass measurement of 120 894 Da indicating the binding of four N monomers to approximately 44 RNA nucleotides (107 004 Da is the expected mass of the tetrameric protein complement only), consistent with the stoichiometry observed by X-ray crystallography.^7^ To test the RNA-induced oligomerisation properties of the BUNV-N protein, it was necessary to remove the bound, endogenous RNA. This was performed by treatment with high ionic strength buffers, as described previously.^7^ ESI-MS was conducted on a sample of high salt-treated BUNV-N (10 μM) after dialysis against a MS-compatible buffer solution (50 mM ammonium acetate, pH 6.8). The spectrum (Fig. 2(b)) displayed a narrow charge state distribution (8+ to 11+) corresponding to a structured species of 26 751 Da in mass, consistent with the sequence mass of monomeric BUNV-N. In addition, low intensity signals at higher *m/z* (*m/z* 5000–7000) were observed which are consistent with tetrameric and pentameric N bound to RNA, the latter of which had survived the RNA-removal step. However, there was no evidence for protein-only oligomers. Together these data suggest that, under these experimental conditions, BUNV-N is unable to form stable higher oligomers. This suggests that the RNA may be required to provide thermodynamic stability to the complex, whilst promoting an oligomerisation-competent conformation of N.

### RNA-free SBV-N exists as monomer, tetramer, and pentamer

For comparison with BUNV-N, ESI-MS was conducted on high salt-treated SBV-N (10 μM, 50 mM ammonium acetate, pH 6.8) (Fig. 2(c)). Monomeric SBV N was observed, as for BUNV-N (measured mass = 26 269 Da), but in this case a significant proportion of the observed signals were attributed to SBV-N complexes in the form of a mixture of tetramers and pentamers (Fig. 2(c)), in contrast to BUNV-N. These data are consistent with previous findings that SBV multimerisation was not wholly dependent on RNA.^7^

### RNA oligonucleotides induce N oligomerisation

**Figure 3:**
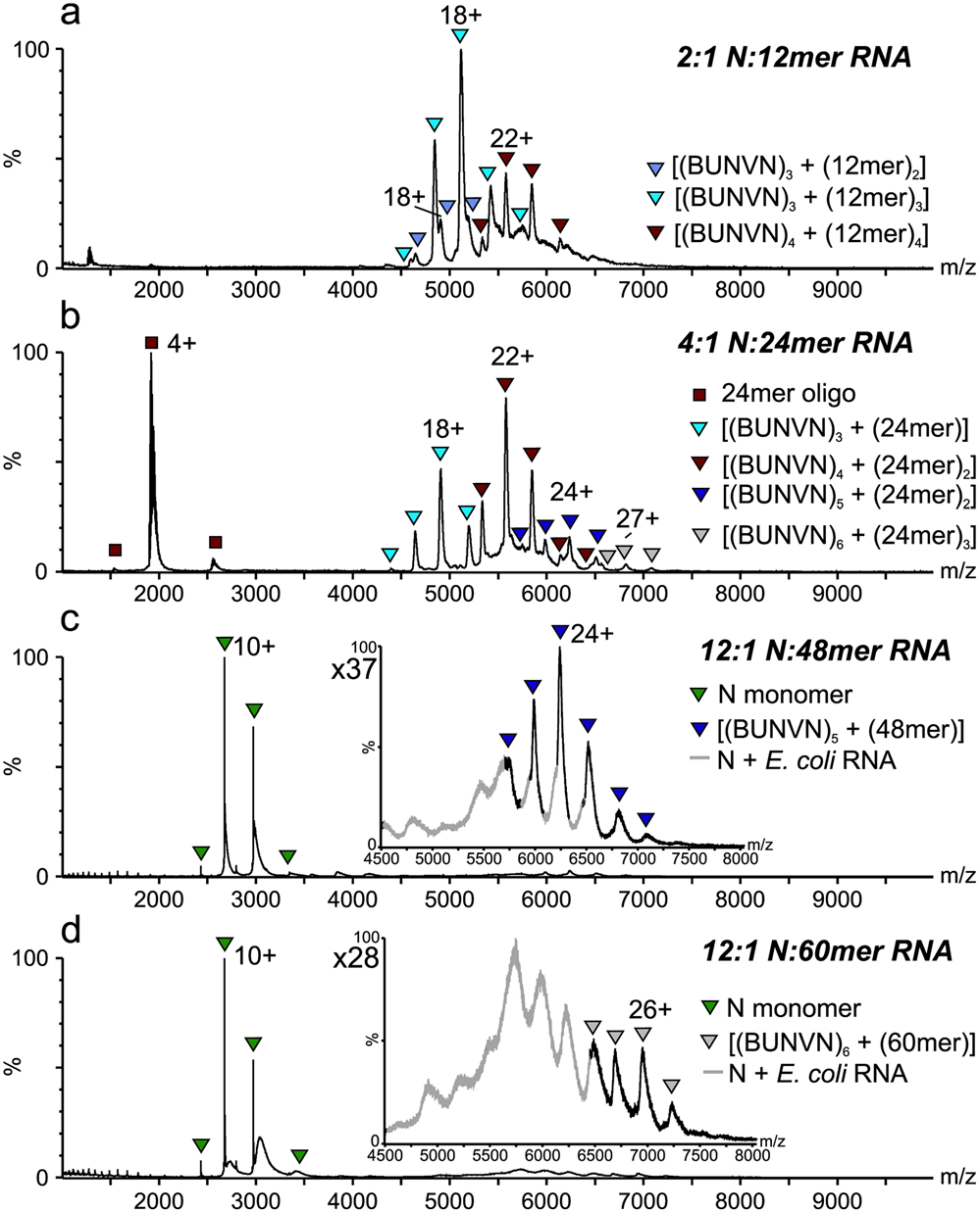
ESI-MS analysis of BUNV-N with added synthetic RNAs. (a) BUNV-N in the presence of the 12-mer RNA forms complexes of [(BUNV)_3_ + (12mer)_2_] (purple triangles), [(BUNV)_3_ + (12mer)_3_] (blue triangles), and [(BUNV)_4_ + (12-mer)_4_] (red triangles); (b) BUNV-N in the presence of the 24-mer RNA forms complexes of [(BUNV)_3_ + (24-mer)] (blue triangles), [(BUNV)_4_ + (24-mer)_2_] (red triangles), [(BUNV)_5_ + (24-mer)_2_] (navy triangles), and [(BUNV)_6_ + (24-mer)_3_] (grey triangles); (c) BUNV-N in the presence of the 48-mer RNA forms complexes of [(BUNV)_5_ + (48-mer)]; inset: expansion of *m/z* 4500–8000 region; (d) BUNV-N in the presence of the 60-mer RNA forms complexes of [(BUNV)_6_ + (60-mer)]; inset: expansion of *m/z* 4500–8000 region. Under both the 48 nt and 60 nt conditions there is a contribution from *E. coli* RNA-bound N (grey signals).

To gain insights into the RNA-binding properties of the N proteins, each of the RNA-free N proteins was incubated with synthetic RNA oligonucleotides (RNAs). The RNA sequences were taken from the 5′ terminus of the BUNV antigenome S segment, in line with the RNA used in our previous fluorescence polarisation assays.^7^ The first 12, 24, 48, and 60 nucleotides were chosen (see Experimental section), based on the assumption that BUNV-N binds around 12 nt per monomer, as suggested by previous data.^24^ BUNV-N was incubated separately with each of the RNAs in a number of molar ratios (Fig. 3 and Table 1). When incubated at a 2:1 (N/RNA) molar ratio with the 12-mer RNA, the resulting mass spectrum displayed signals corresponding to three species: [(BUNVN)_3_ + (12-mer)_2_], [(BUNVN)_3_ + (12-mer)_3_], and [(BUNVN)_4_ + (12-mer)_4_], the latter two being the major species, with no evidence for the monomeric protein (Fig. 3(a)). Firstly, this experiment shows that BUNV-N can be reconstituted into N-RNA oligomeric complexes *in vitro*, in the absence of the cellular environment in which the original *E. coli*-expressed N-RNA tetramers were synthesised. Secondly, the observation of significant quantities of trimers in this spectrum indicates that the tetramer is not the only oligomeric state that BUNV-N–RNA complexes form in solution.

**Table 1:** Summary of protein N–RNA complexes observed during ESI-MS RNA-binding experiments (Figs. 3 and 4) compared with the oligomers observed for the N proteins alone (Fig. 2). The dominant species in each case is shaded

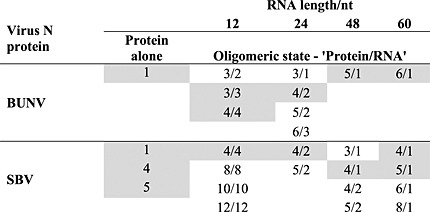

When incubated with the 24 nt RNA (24-mer), signals corresponding to the complexes [(BUNVN)_3_ + (24-mer)_1_], [(BUNVN)_4_ + (24-mer)_2_] (the major species), [(BUNVN)_5_ + (24-mer)_2_], and [(BUNVN)_6_ + (24-mer)_3_] were observed in the mass spectrum (Fig. 3(b)). The observation of pentamers and hexamers is consistent with EM data for BUNV-N from Li *et al*.^10^ which showed evidence for a small number of these species. These data are also supported by studies carried out elsewhere with RVFV-N, for which EM data displayed a wide range of oligomeric states ranging from tetramers to octamers.^25^,^26^

Mixing the 48 nt RNA (48-mer) with BUNV-N gave rise to the pentamer, [(BUNVN)_5_ + (48-mer)_1_], as the dominant species (Fig. 3(c)). In the presence of the longest RNA sequence (60-mer), a low population of the hexamer [(BUNVN)_6_ + (60-mer)_1_] was observed (Fig. 3(d)), which overlapped with low intensity signals corresponding to the *E. coli* RNA-bound BUNV-N carried over from the protein purification, also shown in Fig. 2(a).

**Figure 4:**
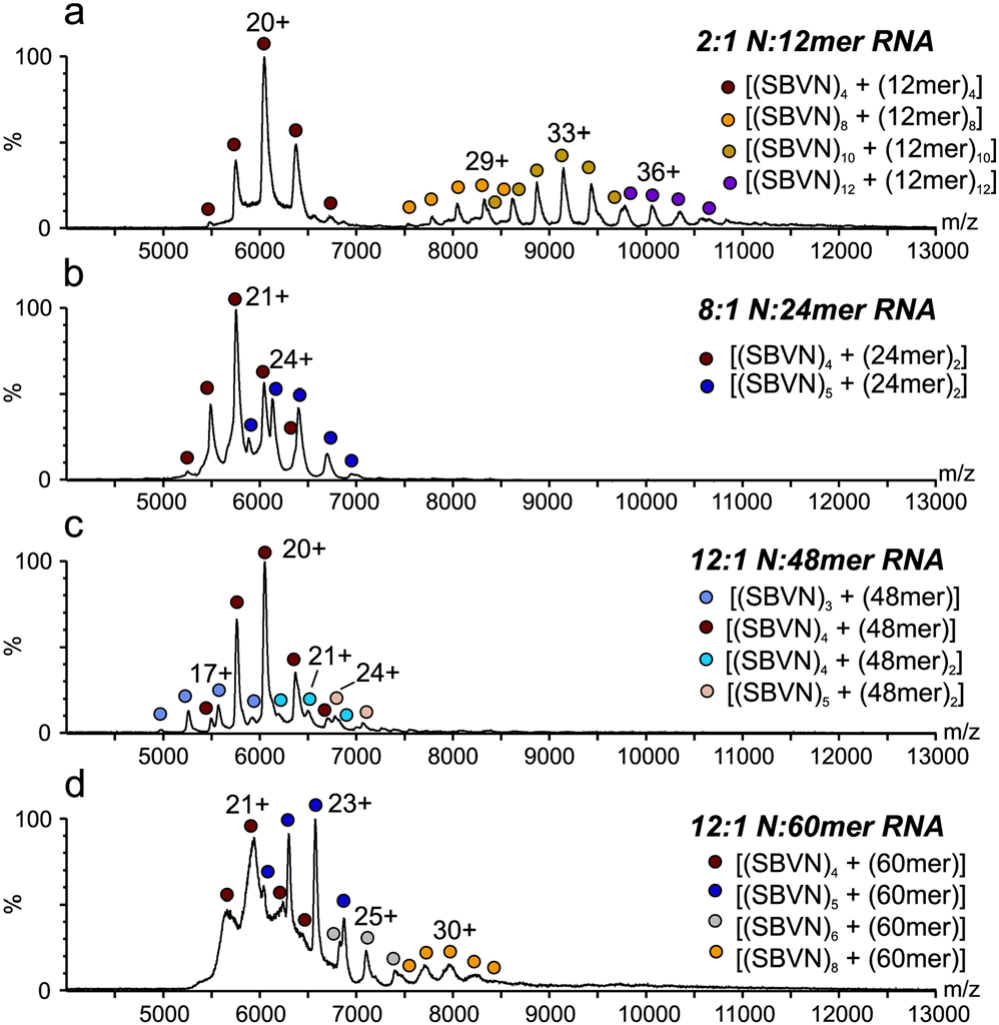
ESI-MS analysis of SBV-N with synthetic RNAs. (a) SBV-N in the presence of the 12-mer RNA forms complexes of [(SBV)_4_ + (12-mer)_4_] (red circles), [(SBV)_8_ + (12-mer)_8_] (yellow circles), [(SBV)_10_ + (12-mer)_10_] (green circles), and [(SBV)_12_ + (12-mer)_12_] (purple circles); (b) SBV-N in the presence of the 24-mer RNA forms complexes of [(SBV)_4_ + (24-mer)_2_] (red circles) and [(SBV)_5_ + (24-mer)_2_] (navy circles); (c) SBV-N in the presence of the 48-mer RNA forms complexes of [(SBV)_3_ + (48-mer)] (purple circles) and [(SBV)_4_ + (48-mer)] (red circles) as well as small amounts of [(SBV)_4_ + (48-mer)_2_] (blue circles) and [(SBV)_5_ + (48-mer)_2_] (beige circles); (d) SBV-N in the presence of the 60-mer RNA forms complexes of [(SBV)_4_ + (60-mer)] (red circles), [(SBV)_5_ + (60-mer)] (navy circles), [(SBV)_6_ + (60mer)] (grey circles), and [(SBV)_8_ + (60-mer)] (yellow circles).

Similar experiments were performed with SBV-N for comparison (Fig. 4 and Table 1) (again using the BUNV S segment antigenome-derived RNAs). Most differences between the two N proteins were observed when SBV N was incubated with the 12-mer RNA. Signals consistent with [(SBVN)_4_ + (12-mer)_4_] (major species observed), [(SBVN)_8_ + (12-mer)_8_], [(SBVN)_10_ + (12-mer)_10_], and [(SBVN)_12_ + (12-mer)_12_] were observed, corresponding to tetramers, octamers, decamers and dodecamers of SBV-N.

In the presence of the 24-mer RNA, SBV-N formed tetrameric and pentameric complexes, [(SBVN)_4_ + (24-mer)_2_] and [(SBVN)_5_ + (24-mer)_2_], respectively (Fig. 4(b)). When incubated with the 48-mer RNA, trimers, tetramers, and pentamers were formed: [(SBVN)_3_ + (48-mer)_1_], [(SBVN)_4_ + (48-mer)_1_] (major species observed), [(SBVN)_4_ + (48-mer)_2_], and [(SBVN)_5_ + (48-mer)_2_] (Fig. 4(c)). In the presence of the 60-mer RNA, SBV-N shows evidence of tetramers, pentamers, hexamers, and octamers with stoichiometries of [(SBVN)_4_ + (60-mer)_1_] (equal major species observed), [(SBVN)_5_ + (60-mer)_1_] (equal major species observed), [(SBVN)_6_ + (60-mer)_1_], and [(SBVN)_8_ + (60-mer)_1_], respectively (Fig. 4(d)). In the case of the [(SBVN)_8_ + (60-mer)_1_] complex, some of the SBV-N monomers cannot be in contact with RNA, which is consistent with SBV-N being able to oligomerise without RNA (Fig. 2(c)), in contrast to BUNV-N.

The preferred oligomerisation state of the SBV-N protein in all experiments was the tetramer, in complex with varying molecules of RNA, in contrast to BUNV-N. Furthermore, the SBV-N protein was detected in a wider range of complexes than the BUNV-N protein, although some of these may have arisen from the presence of the protein-only complexes observed in the absence of RNA (Fig. 2(c)).

### Orthobunyvirus N has a minimal RNA-binding region to activate oligomerisation

In addition to illustrating the diversity in the range of low oligomeric states formed by BUNV-N and SBV-N, these mass spectrometric data also provide insights into the nature of RNA binding. From the X-ray crystal structures of orthobunyavirus N,^7^,^9^–^12^ it was shown that 11 nt were bound to a single N molecule in the tetramer. This is largely consistent with the observations here, but there are some interesting exceptions. Specifically, when BUNV-N is incubated with the 12-mer or 24-mer RNAs, the complexes [(BUNVN)_3_ + (12-mer)_2_] and [(BUNVN)_3_ + (24-mer)_1_], respectively, are observed. In these trimeric complexes there are 33 RNA base binding sites but only 24 are filled, leaving 9 unoccupied. It follows that not all available sites have to be filled in order for oligomerisation to occur, suggesting that there is a minimal region on the protein sensitive to RNA binding that induces oligomerisation. The observation of the complexes [(BUNVN)_5_ + (24-mer)_2_], [(BUNVN)_6_ + (24-mer)_3_], [(BUNVN)_5_ + (48-mer)_1_], and [(BUNVN)_6_ + (60-mer)_1_] in the spectra is also consistent with the accepted protein-RNA stoichiometry of 11 nt per monomer, but again there must be some empty sites. The observation of such a wide range of stoichiometries in these data raises the question as to the structure of these higher oligomers of N when in complex with RNA. The tetramers are likely to be planar ring structures, as supported by X-ray crystallography and EM.^7^ In order to elucidate the dimensions of the oligomers, rotationally averaged collision cross-sectional areas were estimated from ion mobility spectrometry-MS experiments (data not shown). However, it was not possible to differentiate between stacked ring and helical structures as the differences in the modelled collision cross-sectional areas were too small to be differentiated between, although both were within the error of the theoretical modelling methods employed. Ion mobility spectrometry-MS data do, however, rule out single-ring structures for the octamers, decamers and dodecamers of SBV-N, which exhibited collision cross-sectional areas consistent with more compact architectures (Supplementary Table S1, see Supporting Information). The dominance of compact structures suggests that the complexes generated here recapitulate the condensed RNP form observed by EM.^7^,^12^

### The importance of dimers

With tetramers being the dominant oligomeric state observed for SBV-N and one of the most highly populated complexes observed for BUNV-N, one might have expected to observe dimers, especially since in the presence of the 24-mer RNA, there are approximately the correct number of bases to fill all sites (22 sites in total) on the protein. However, there was no trace of dimeric complexes for SBV-N or BUNV-N, in the presence or absence of RNA. Seemingly in conflict with this observation, the higher oligomers of the N proteins varied by two subunits (no odd-numbered oligomers were detected above the size of pentamers), implying that the dimer may indeed be an important structural building block. Together, these observations suggest that the dimeric form of N is transient and consumed on a timescale much faster than can be resolved by MS.

### Despite similarities, SBV-N and BUNV-N do not interact

**Figure 5:**
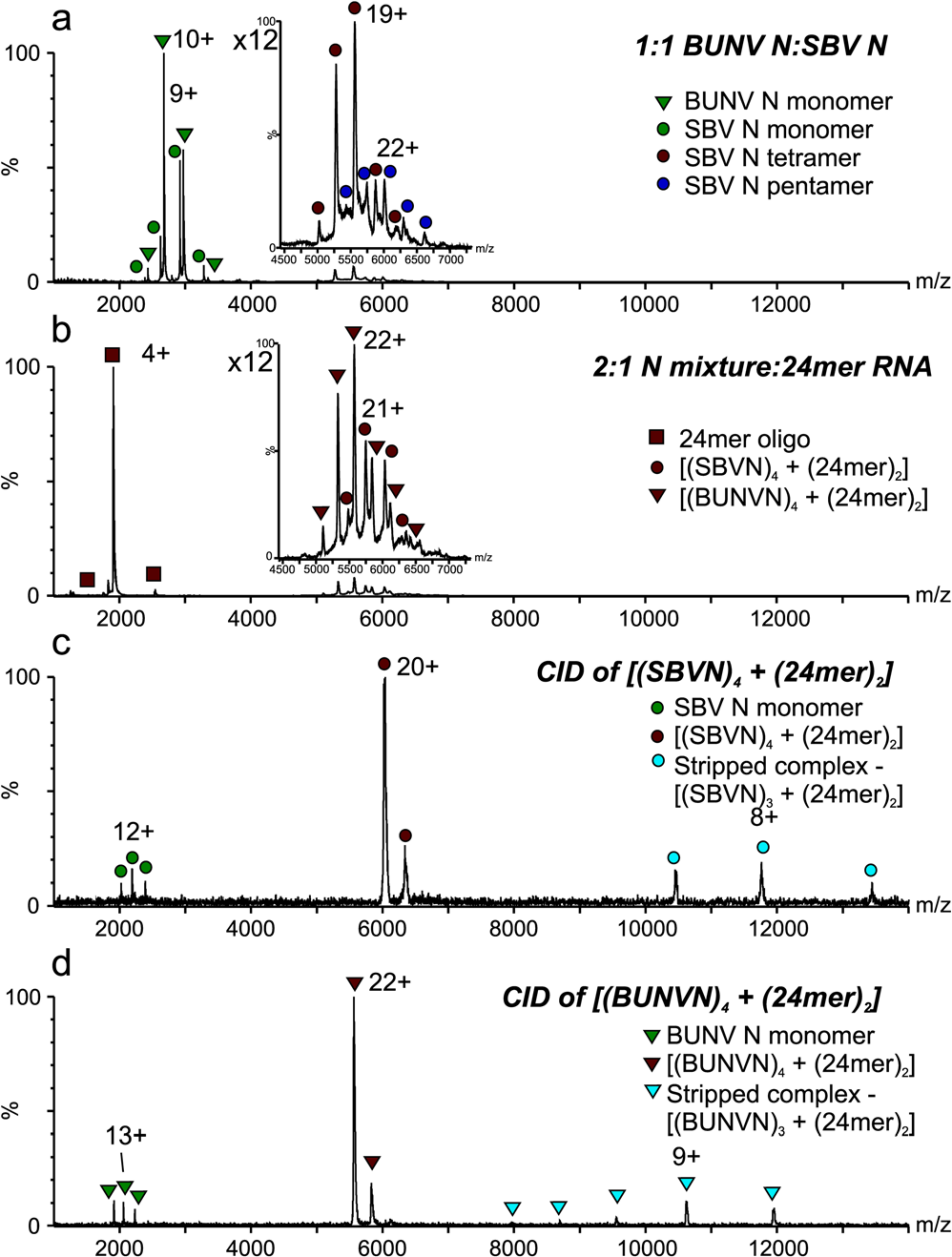
ESI-MS shows that oligomerisation is cooperative between RNA and protein. (a) RNA-free SBV-N tetramers (red circles) and pentamers (navy circles) are not disrupted by possible competing interactions with BUNV-N (monomers, green triangles), suggesting incompatible N-N interfaces, inset: expansion of *m/z* 4500–7000 region; (b) 1:1 molar BUNV-N: SBV-N in the presence of the 24-mer RNA (red squares, 2:1 total protein:RNA). The resulting RNA-protein complexes are identified as [(BUNV)_4_ + (24-mer)_2_] (red triangles) and [(SBV)_4_ + (24-mer)_2_] (red circles), inset: expansion of *m/z* 4500–7000 region; (c) CID MS/MS of the 20+ charge state ions of [(SBV)_4_ + (24-mer)_2_] (red circles). Only the expected SBV-N monomer (11+ to 13+) (green circles) and trimeric stripped complex ions (7+ to 9+) (blue circles) are observed; (d) CID MS/MS of the 22+ charge state ions of [(BUNV)_4_ + (24-mer)_2_] (red triangles). Only the expected BUNV-N monomer (12+ to 14+) (green triangles) and stripped trimeric complex ions (8+ to 12+) (blue triangles) are observed. Thus, the MS/MS data verify the MS data in (b).

BUNV-N and SBV-N have been shown to have very similar three-dimensional structures,^7^–^10^ yet only 37% sequence identity (Fig. 1(c)). It has been proposed also that the N- and C-terminal arms are the major driving force for the protein-protein interactions that give rise to oligomerisation. To test this theory and to determine whether the oligomerisation of the N proteins can be driven entirely by the RNA, the two N proteins were mixed in a 1:1 molar ratio and analysed in the presence and absence of RNA (in this case the 24-mer) (Fig. 5). We explored the possibility that, in the presence of RNA-free monomeric BUNV-N, the RNA-free SBV-N tetramers and pentamers may be disrupted by competing interactions, leading to mixed N protein complexes. When the two proteins were mixed in a 1:1 molar ratio in the absence of RNA, we did not detect any such mixed complexes using ESI-MS analysis, only intact SBV-N tetramers and pentamers together with monomeric SBV-N and BUNV-N (Fig. 5(a)). This is consistent with our earlier observations when analysing both proteins separately (Figs. 2(a) and 2(b)). To investigate whether BUNV-N and SBV-N could bind simultaneously to a single RNA molecule, 24-mer RNA was added to the protein mixture (2:1, N to RNA). Signals corresponding to N protein–RNA complexes were observed after mixing. From mass measurements alone, the dominant complexes were identified as the homomeric species [(BUNVN)_4_ + (24-mer)_2_] and [(SBVN)_4_ + (24-mer)_2_], indicating that no mixed complexes had been formed, and also the preferred tetrameric complexes with stoichiometries of 12 nucleotides per protein molecule were observed for both BUNV-N and SBV-N, whether analysed alone or within a mixture (Fig. 5(b)). The stoichiometries of the two complexes were verified unambiguously using MS/MS with CID. MS/MS of the 20+ charge state ions of the [(SBVN)_4_ + (24-mer)_2_] complex yielded signals corresponding to SBV-N monomers and the charge-reduced 'stripped' complex [(SBVN)_3_ + (24-mer)_2_], as expected (Fig. 5(c)). Similarly, MS/MS of the 22+ charge state ions of the [(BUNVN)_4_ + (24-mer)_2_] complexes yielded signals corresponding to BUNV-N monomers and the 'stripped' complex [(BUNVN)_3_ + (24-mer)_2_] (Fig. 5(d)).

The crystal structures of the N proteins from BUNV and SBV, as well as those of the other orthobunyaviruses LACV and LEAV, indicate that the protein-protein N-N interface is dominated by the N- and C-terminal arms.^7^–^12^ This raises the question of which part of the protein sequence exerts specificity? On inspection of the N-terminal sequence of the N proteins of the four orthobunyaviruses BUNV, SBV, LACV, and LEAV, it is apparent that this region is less conserved than the C-terminus (Fig. 1(c)), which therefore may indicate that the incompatibility between N proteins is dominated by the N-terminal sequence.

Another possible reason for the absence of mixed complexes is the greater affinity of SBV-N for ssRNA, as demonstrated previously by fluorescence polarisation assays,^7^ but as the assays show that there is less than a factor of three between their relative affinities, RNA binding is unlikely to be the sole factor in the discrimination of the N proteins.

Furthermore, these data suggest some general properties of N oligomerisation: that the oligomerisation process is highly cooperative between the protein and RNA and is not a simple 'beads-on-a-string' polymerisation mechanism. If the oligomerisation process was driven predominantly by the electrostatic interactions between N and the RNA backbone, one might expect to see heteromeric N oligomers as conceivably both proteins can bind to the same piece of RNA. However, the data are consistent with RNA-induced protein-protein recognition so fine-tuned that the relatively similar structures of BUNV-N and SBV-N cannot interact, resulting in no evidence for mixed complexes. A possible oligomerisation mechanism would involve the N proteins condensing onto the RNA, subsequently promoting a conformation that induces further N binding. When both N proteins were mixed, it would be at this point that they discriminate between each other and if any proteins condensed on the same RNA, homomeric interactions would out-compete heteromeric ones.

## CONCLUSIONS

This work constitutes the first noncovalent mass spectrometry study of viral proteins from the *Bunyaviridae* family, focusing on the N proteins of the prototypical Bunyamwera virus, and the recently emerging ruminant livestock-infecting Schmallenberg virus. The data have demonstrated that despite their similarity in monomer structure, BUNV-N and SBV-N form unique ranges of oligomers upon binding to RNA. The data have also provided insights into the RNA-binding and oligomerisation properties of orthobunyavirus N proteins.

The detection of a range of stoichiometries for both BUNV-N and SBV-N in the presence of added RNA suggests that the tetrameric form of N (observed by crystallography, size-exclusion chromatography, analytical ultracentrifugation, EM and MS) is not the sole building block of the orthobunyavirus RNP, consistent with EM images of the native RNPs and a crystal form showing a helical geometry.^12^ The lack of observed dimers but the favoured, but not exclusive, formation of even-numbered oligomers may implicate dimers as an important structural building block, crucial for oligomerisation. The range of oligomeric states observed for the N proteins is consistent with flexibility in the N- and C-terminal arms that constitute the protein-protein interaction interface, and supports the observation of both condensed and relaxed RNP forms.

The oligomerisation process is highly cooperative between protein and RNA and is not dominated by the electrostatic interactions between N proteins and the phosphate backbone, but likely involves protein condensation onto the RNA followed by the conformational promotion of oligomer growth. As not all nucleotide-binding sites on N proteins need to be occupied in order to promote this oligomerisation, a minimal RNA-sensitive binding region is likely. This leads to a model reminiscent of the assembly of tobacco mosaic virus via formation of a split ring (or lockwasher) of protein subunits in order to generate a nucleus for addition of further subunits to form a helical RNP complex.^27^ This work demonstrates the use of mass spectrometry in the analysis of the oligomerisation and RNA-binding properties of the N proteins of these emerging viruses. The detailed information obtained from MS experiments on stoichiometries and oligomeric states complements X-ray crystallographic and EM data and thus makes ESI-IMS an important technique for studies of these assemblies, furthering the structural understanding of the viruses, which is key to the development of effective therapeutics.

## Supporting Information

Supporting info item
