# Supplementary material for: Probing Bunyavirus N protein oligomerisation using mass spectrometry
Source: Rapid Commun Mass Spectrom. 2014 Feb 17;28(7):793–800. doi: 10.1002/rcm.6841 (PMC4377080; doi:10.1002/rcm.6841)
Supplement: Supplementary file 1 — Supporting info item [file rcm0028-0793-sd1.docx]

# Probing Bunyavirus N protein oligomerisation using mass spectrometry

Dale A. Shepherd^#^, Antonio Ariza, Thomas A. Edwards, John N. Barr, Nicola J. Stonehouse*, Alison E Ashcroft*

Astbury Centre for Structural Molecular Biology, Faculty of Biological Sciences,

University of Leeds, Leeds, LS2 9JT, UK.

**SUPPLEMENTARY INFORMATION**

Table S1. Table of experimental and theoretical collision cross-sectional areas (CCS) for SBV-N-RNA complexes illustrating the compactness of the higher oligomers. The theoretical values in green show good agreement with experiment.

n.b. The experimental tetramer value was used during optimisation of coarse-grained models, and so necessarily agrees with the theoretical value.

| **SBV N oligomer** | **Experimental CCS / nm^2^** | **Theoretical single-ring CCS / nm^2^** | **Theoretical double-ring (compact) CCS / nm^2^** |
| --- | --- | --- | --- |
| 3 | 45.7 ± 2.3 | 46.6 | N/A |
| 4 | 56.4 ± 0.2 | 56.5 | N/A |
| 5 | 67.2 ± 0.2 | 68.3 | N/A |
| 6 | 77.3 ± 0.2 | 79.9 | N/A |
| 8 | 94.0 ± 0.7 | 105 | 94.5 |
| 10 | 113 ± 1 | 129 | 112 |
| 12 | 128 ± 2 | 153 | 129 |
